# Supplementary material for: Deep inspiration breath hold versus free breathing in postoperative radiotherapy strategy for patients with left-sided breast cancer treated with volumetric modulated arc therapy: A meta-analysis and systematic review
Source: PLoS One. 2026 Mar 30;21(3):e0345614. doi: 10.1371/journal.pone.0345614 (PMC13035160; doi:10.1371/journal.pone.0345614)
Supplement: Attachment — Submitted filename: Response To Reviewers.docx [file pone.0345614.s002.docx]

Response to Reviewers

Manuscript ID: PONE-D-24-45601
Title: Deep inspiration breath hold versus free breathing in postoperative radiotherapy strategy for patients with left-sided breast cancer treated with volumetric modulated arc therapy: A meta-analysis and systematic review

We thank the Academic Editor and the Reviewers for their thoughtful and constructive comments on our manuscript. We have carefully addressed each of the points raised and revised the manuscript accordingly. Below are our point-by-point responses to the reviewers’ comments.

**Reviewer #1**

**Methods:**

1. **Search engine selection:**
   Thank you for your valuable feedback regarding the literature search strategy.
   We acknowledge the overlap between PubMed and EMBASE, which may account for the number of duplicates identified. While we considered the inclusion of additional databases such as Web of Science or Google Scholar, we ultimately limited our search to PubMed, EMBASE, and the Cochrane Library. This approach was based on the recommendations outlined by the Oxford Centre for Evidence-Based Medicine (CEBM), which supports the use of these databases as core sources for systematic reviews and evidence synthesis.
   Our decision was also made to maintain methodological consistency and ensure the use of databases with robust indexing and reproducibility standards. While we recognize that other platforms like Google Scholar may offer broader retrieval, they may also include non-peer-reviewed or non-indexed literature, which could introduce variability and limit replicability.
   We believe that our selected databases provide a comprehensive and focused representation of the current evidence in the field. Nevertheless, we sincerely appreciate your suggestion and will take it into account in future work.
2. **Sensitivity analyses detail:**
   Thank you for your insightful comment regarding the sensitivity analyses.
   In our study, we conducted several sensitivity tests to evaluate the robustness of the primary findings. Specifically, we applied the following approaches:

- Exclusion of high-risk studies.
- Use of both fixed-effect and random-effects models.
- Leave-one-out analysis.
- Limiting to RCTs only.
- Subgroup-specific exclusions by region.
  We have now added this clarification to the revised manuscript under the Methods section. We appreciate your suggestion, which helped improve the clarity of our methodology.

3. **Exclusion criteria wording:**
   Thank you for pointing this out.
   We agree with your observation that “failure to meet the inclusion criteria” is redundant as an exclusion criterion, since it is inherently implied. Accordingly, we have revised the exclusion criteria list in the manuscript to remove this item and improve clarity.
   We appreciate your helpful suggestion in refining the methodology section.
4. **Search strings inclusion:**
   Thank you for your helpful suggestion.
   In response, we have included the detailed search strategies used for PubMed, Cochrane Library, and Embase as a supplementary file. The search terms combined controlled vocabulary (MeSH/Emtree terms) and relevant keywords to enhance both precision and sensitivity of the search. This addition improves the transparency and reproducibility of our review process, and we appreciate your guidance on strengthening the methodological reporting.
5. **Quality assessment details:**
   Thank you for your insightful comment.
   We have clarified the quality assessment process in the revised manuscript. Specifically, two reviewers (PYC and PJH) independently conducted the quality assessment and data extraction using a standardized form. In case of discrepancies, consensus was sought through discussion, and unresolved issues were decided by a third reviewer (CCC). This approach ensured reliability and consistency.

**Results:**

1. **Table 2 – staging and pathology:**
   Thank you for your comment. Most of the included studies were dosimetric planning analyses and did not report detailed pathology or staging information. We have added the available staging data to Table 2 for clarity. The existing “Treated area” column also provides relevant context on disease extent that may influence dosimetric outcomes.
2. **Use of “NR” and SD for age:**
   We appreciate the suggestion. “Not mentioned” has been replaced with “NR” in Table 1 and 2. Standard deviations for mean age have also been included where available; only Xu (2023) reported this information.
3. **Figure quality:**
   Thank you for your valuable feedback regarding the figure quality.
   We have revised Figures 2 through 7 by improving their layout and increasing the resolution from 300 dpi to 600 dpi. All figures have been saved as high-quality TIFF files to enhance clarity and legibility for better interpretation of the findings.

**Discussion/Conclusion:**

1. **Context of VMAT vs. 3D-CRT:**
   Thank you for your insightful comment. As suggested, we have expanded the Introduction to include background on VMAT, its technical characteristics, and clinical considerations compared to 3D-CRT. We also revised this section to clarify that previous meta-analyses included mixed radiotherapy techniques, which may have confounded technique-specific interpretations. In contrast, our study focuses exclusively on VMAT combined with DIBH, providing a more targeted and meaningful analysis.
2. **Writing error (line 286):**
   Thank you for highlighting this point. We have corrected the typographical error at line 286 by removing the underscore symbol and adjusted the wording to clarify the strength of evidence at line 327.
3. **Subgroup analysis and VMAT advantages:**
   Thank you for your thoughtful comment. We have revised the discussion to further elaborate on the potential advantages of VMAT and to emphasize that, based on our subgroup analysis, the heart-sparing effect of DIBH was consistently observed across different clinical scenarios. This suggests the robustness and clinical applicability of combining VMAT with DIBH in left-sided breast cancer treatment.
4. **Separate paragraph for limitations:**
   Thank you for the valuable suggestion. In response, we have added a separate paragraph in the Discussion to address the study’s limitations, including the retrospective nature of the included studies, small sample sizes, potential anatomic variability, and limited database scope. While acknowledging that similar meta-analyses exist, we emphasized that our study provides a more focused and detailed evaluation of DIBH specifically within the VMAT setting.

**Reviewer #2**

1. **Line 84 – Overstatement about VMAT heart dose:**
   Thank you for your valuable feedback. We acknowledge that the original statement was overly strong and may have misrepresented the comparative dosimetric profiles of VMAT, IMRT, and 3D-CRT. In response, we have revised the paragraph in the Introduction to clarify that while VMAT may offer dosimetric advantages in certain complex clinical settings—such as in cases requiring comprehensive nodal irradiation—its use may also result in increased low-dose exposure to surrounding normal tissues. This provides a more balanced and accurate description, as suggested.
2. **Line 295 – Clarification about V20:**
   Thank you for the correction (line 336). We have revised the sentence to clarify that V20 refers to the volume receiving 20 Gy, and not a mean dose.
3. **Breathing pattern and lung dose variation:**
   Thank you for your comments. In response, we added a statement in the Discussion section noting that differences in breathing patterns (abdominal vs. thoracic) may influence DIBH effectiveness. However, none of the included studies reported this information.
4. **Coaching and DIBH program implementation:**
   Thank you for this insightful suggestion. In response, we have added a paragraph at the end of the Discussion section to highlight key considerations for implementing and coaching DIBH. This includes referencing the ESTRO-ACROP guideline, which provides recommendations on equipment, staff training, patient communication, and breath-hold coaching strategies.

We sincerely thank both reviewers for their thorough evaluation and constructive feedback, which greatly improved the clarity and quality of our manuscript.

Sincerely,
The Corresponding Authors
On behalf of all authors
